# Two-year efficacy and safety of risdiplam in patients with type 2 or non-ambulant type 3 spinal muscular atrophy (SMA)

**DOI:** 10.1007/s00415-023-11560-1

**Published:** 2023-02-03

**Authors:** Maryam Oskoui, John W. Day, Nicolas Deconinck, Elena S. Mazzone, Andres Nascimento, Kayoko Saito, Carole Vuillerot, Giovanni Baranello, Nathalie Goemans, Janbernd Kirschner, Anna Kostera-Pruszczyk, Laurent Servais, Gergely Papp, Ksenija Gorni, Heidemarie Kletzl, Carmen Martin, Tammy McIver, Renata S. Scalco, Hannah Staunton, Wai Yin Yeung, Paulo Fontoura, Eugenio Mercuri, on behalf of The SUNFISH Working Group

**Affiliations:** 1 Departments of Pediatrics and Neurology and Neurosurgery, McGill University, Montreal, Canada; 2 Department of Neurology, Stanford University, Palo Alto, CA USA; 3 Neuromuscular Reference Center, UZ Gent, Ghent, Belgium; 4 Centre de Référence des Maladies Neuromusculaires et Service de Neurologie Pédiatrique, Queen Fabiola Children’s University Hospital, Université Libre de Bruxelles, ULB, Brussels, Belgium; 5 Pediatric Neurology Institute, Catholic University and Nemo Pediatrico, Fondazione Policlinico Gemelli IRCCS, Rome, Italy; 6 Neuromuscular Unit, Neuropaediatrics Department, Hospital Sant Joan de Déu, Fundacion Sant Joan de Deu, CIBERER–ISC III, Barcelona, Spain; 7 Institute of Medical Genetics, Tokyo Women’s Medical University, Tokyo, Japan; 8 Department of Pediatric Physical Medicine and Rehabilitation, Hôpital Mère Enfant, CHU-Lyon, Lyon, France; 9 Neuromyogen Institute, CNRS UMR 5310-INSERM U1217, Université de Lyon, Lyon, France; 10 The Dubowitz Neuromuscular Centre, NIHR Great Ormond Street Hospital Biomedical Research Centre, Great Ormond Street Institute of Child Health, University College London and Great Ormond Street Hospital Trust, London, UK; 11 Developmental Neurology Unit, Fondazione IRCCS Istituto Neurologico Carlo Besta, Milan, Italy; 12 Neuromuscular Reference Centre, Department of Paediatrics and Child Neurology, University Hospitals Leuven, Leuven, Belgium; 13 Department of Neuropediatrics and Muscle Disorders, Faculty of Medicine, Medical Center–University of Freiburg, Freiburg, Germany; 14 Department of Neurology, Medical University of Warsaw, Warsaw, Poland; 15 I-Motion-Hôpital Armand Trousseau, Paris, France; 16 MDUK Oxford Neuromuscular Centre, Department of Paediatrics, University of Oxford, Oxford, UK; 17 Division of Child Neurology, Centre de Références des Maladies Neuromusculaires, University Hospital Liège and University of Liège, Liège, Belgium; 18 Pharma Development, Safety, F. Hoffmann-La Roche Ltd, Basel, Switzerland; 19 PDMA Neuroscience and Rare Disease, F. Hoffmann-La Roche Ltd, Basel, Switzerland; 20 Roche Pharmaceutical Research and Early Development, Roche Innovation Center Basel, Basel, Switzerland; 21 Roche Products Ltd, Welwyn Garden City, UK; 22 Pharma Development Neurology, F. Hoffmann-La Roche Ltd, Basel, Switzerland

**Keywords:** Spinal muscular atrophy, Risdiplam, SMA, SUNFISH, Motor function, Safety

## Abstract

Risdiplam is an oral, survival of motor neuron 2 (*SMN2*) pre-mRNA splicing modifier approved for the treatment of spinal muscular atrophy (SMA). SUNFISH (NCT02908685) Part 2, a Phase 3, randomized, double-blind, placebo-controlled study, investigated the efficacy and safety of risdiplam in type 2 and non‑ambulant type 3 SMA. The primary endpoint was met: a significantly greater change from baseline in 32-item Motor Function Measure (MFM32) total score was observed with risdiplam compared with placebo at month 12. After 12 months, all participants received risdiplam while preserving initial treatment blinding. We report 24-month efficacy and safety results in this population. Month 24 exploratory endpoints included change from baseline in MFM32 and safety. MFM‑derived results were compared with an external comparator. At month 24 of risdiplam treatment, 32% of patients demonstrated improvement (a change of ≥ 3) from baseline in MFM32 total score; 58% showed stabilization (a change of ≥ 0). Compared with an external comparator, a treatment difference of 3.12 (95% confidence interval [CI] 1.67–4.57) in favor of risdiplam was observed in MFM-derived scores. Overall, gains in motor function at month 12 were maintained or improved upon at month 24. In patients initially receiving placebo, MFM32 remained stable compared with baseline (0.31 [95% CI – 0.65 to 1.28]) after 12 months of risdiplam; 16% of patients improved their score and 59% exhibited stabilization. The safety profile after 24 months was consistent with that observed after 12 months. Risdiplam over 24 months resulted in further improvement or stabilization in motor function, confirming the benefit of longer-term treatment.

**Supplementary Information:**

The online version contains supplementary material available at 10.1007/s00415-023-11560-1.

## Introduction

### Overview of SMA and the natural history of types 2 and 3 SMA

Spinal muscular atrophy (SMA) is an autosomal recessive neuromuscular disorder caused by reduced levels of survival of motor neuron (SMN) protein due to homozygous deletions or loss-of-function mutations in the *SMN1* gene [1]. A homologous gene, *SMN2*, produces only low levels of functional SMN protein due to alternative splicing of its pre-mRNA that excludes exon 7 from the majority of its transcript [1, 2]. Thus, *SMN2* is unable to compensate for the loss of *SMN1* [3].

Patients with type 2 SMA develop symptoms between 6 and 18 months of age, achieve the ability to sit independently and occasionally stand or take a few steps with support, but are unable to walk independently [1, 4]. Those with type 3 SMA experience symptom onset after 18 months of age and achieve the ability to walk independently, although this ability may be lost [1, 5]. The type 2 and 3 SMA patient population is broad and includes children, teenagers and adults with varied functional statuses, contractures, and scoliosis.

### Risdiplam overview

Risdiplam (Evrysdi® [F. Hoffmann La-Roche Ltd/Genentech Inc.]) is an orally administered small molecule indicated for the treatment of patients in the USA [6] and for the treatment of patients with SMA aged ≥ 2 months with type 1, 2, or 3 SMA and one to four copies of the *SMN2* gene in the EU [7]. The label indication in the USA was expanded in 2022 to include patients < 2 months of age, based on interim efficacy and safety data from the RAINBOWFISH (NCT03779334) study showing that pre-symptomatic babies reached key motor milestones after 12 months of risdiplam treatment [8].

Risdiplam modifies *SMN2* pre-mRNA splicing to promote the inclusion of exon 7 and increase levels of functional SMN protein [9]. In SMA mouse models, risdiplam treatment led to a robust increase in functional SMN protein in the central nervous system and in peripheral tissues [10, 11]. The efficacy of risdiplam has been demonstrated in infants with type 1 SMA [12] and in individuals with type 2 and type 3 SMA [13, 14].

### Overview of the SUNFISH study

SUNFISH (NCT02908685) [15] is an ongoing, multi-center, randomized, double-blind, placebo-controlled, two-part, Phase 2/3 study that assessed the efficacy, safety, tolerability, pharmacokinetics, and pharmacodynamics of risdiplam in a broad patient population of children, teenagers, and adults aged 2–25 years with type 2 or type 3 SMA; the study did not exclude patients with low baseline motor function or hallmarks of more advanced disease, such as severe scoliosis, contractures, impaired bulbar function, and a need for enteral feeding or non-invasive ventilation.

Part 1 was a dose-finding study in patients with type 2 or type 3 SMA (ambulant and non‑ambulant) to inform the dose for Part 2. In Part 1, risdiplam treatment led to a sustained increase in SMN protein in the blood, and exploratory efficacy analyses showed improvement or stabilization in motor function [14]. Confirmatory Part 2 investigated the efficacy of risdiplam in individuals with type 2 or non-ambulant type 3 SMA at the dose selected in Part 1. SUNFISH Part 2 met the primary endpoint, demonstrating a statistically significant difference between patients treated with risdiplam and those treated with placebo in the change from baseline in the 32-item Motor Function Measure (MFM32) total score at month 12 [13].

Here, we report longer-term exploratory efficacy and safety results after 24 months of risdiplam treatment in SUNFISH Part 2 and contextualize these findings with external comparator groups. In addition, we investigate the efficacy and safety of 1 year of risdiplam treatment in patients who previously received placebo up to month 12.

## Methods

### Study oversight

This trial was approved by an ethics committee at each study site and was conducted in accordance with Good Clinical Practice guidelines and the World Medical Association Declaration of Helsinki. Written informed consent was provided by the patient or by parents/caregivers. The sponsor, F. Hoffmann-La Roche Ltd, provided the study drug, study management and medical monitoring, drug safety management and analysis, data management and statistical analysis, and pharmacokinetic/pharmacodynamic analysis. Confidentiality agreements were in place between the authors and F. Hoffmann-La Roche Ltd. An external independent data monitoring committee monitored the safety of patients. All authors attest to adherence to the protocol, accuracy of analysis, and complete reporting of adverse events (AEs).

### Patients and study procedures

Eligible patients were non-ambulant and aged 2–25 years, with a genetically confirmed diagnosis of 5q-autosomal recessive SMA and clinical symptoms attributable to type 2 or type 3 SMA. Patients were excluded from the study if they had received treatment with an *SMN2*-targeting antisense oligonucleotide, *SMN2* splicing modifier, or gene therapy. Full inclusion and exclusion criteria have been published previously [13].

### Study design and outcomes

Patients were stratified by age (2–5, 6–11, 12–17, and 18–25 years) and randomized 2:1 with concealed allocation to receive either risdiplam or placebo daily for 12 months. The risdiplam dose was 0.25 mg/kg for patients weighing < 20 kg, and 5 mg for patients weighing ≥ 20 kg [13]. After 12 months, patients receiving placebo were switched to risdiplam in a blinded manner (i.e., at their week 52 visit) and all patients were treated with risdiplam until month 24.

The 24-month exploratory objectives and outcomes included the efficacy of risdiplam treatment with regard to motor function (as measured by MFM32 [16], Hammersmith Functional Motor Scale—Expanded [HFMSE] [17], and Revised Upper Limb Module [RULM] [18]); respiratory function (as measured by sniff nasal inspiratory pressure, maximal inspiratory pressure, maximal expiratory pressure, forced vital capacity [FVC], forced expiratory volume in the first second, and peak cough flow); patient- and caregiver-reported independence (as measured by the SMA Independence Scale-Upper Limb Module [SMAIS-ULM] [19]); and safety and tolerability. The scoring methods for each endpoint in this study have been described previously [13].

Safety was assessed throughout the study by monitoring and recording AEs, including serious AEs (SAEs), laboratory assessments, electrocardiograms, vital signs, and ophthalmologic, neurologic, and anthropometric examinations.

### Statistical methods

Exploratory efficacy analyses were conducted based on the all-exposure-to-risdiplam treatment period (the treatment period after receiving the first dose of risdiplam). Randomized patients who did not receive risdiplam treatment were not included in the exploratory efficacy analyses; this condition applied to one patient who was originally randomized to the placebo group but left the study early, and thus did not receive risdiplam treatment. For each efficacy endpoint, individuals who fulfilled the corresponding missing motor function scale item rules were excluded at the corresponding time point, as predefined in the statistical analysis plan.

Efficacy endpoints were summarized by randomized treatment (risdiplam or placebo switched to risdiplam) for the all-exposure-to-risdiplam treatment period. For patients initially on risdiplam treatment, results at month 12, month 18, and month 24 were summarized. For patients initially on placebo, the adjusted baseline (defined as the last measurement prior to the first dose of risdiplam) was used for the analyses and results at month 12 on risdiplam treatment (i.e., month 24 in the study) were summarized.

All patients who received at least one dose of risdiplam (*n* = 120) or placebo (*n* = 60) were included in the safety-evaluable population. Safety data were summarized descriptively by treatment group for the first 12-month placebo-controlled period (i.e., 0–12 months of treatment for the risdiplam group), and for the open-label treatment period (i.e., 12–24 months of risdiplam treatment for the risdiplam group and 0–12 months of risdiplam treatment for patients who previously received placebo).

After month 12, all patients in SUNFISH received risdiplam while maintaining blinding to the initial treatment randomization. An external comparator group of untreated individuals with Type 2 and Type 3 SMA was, therefore, used to give context to SUNFISH Part 2 results at month 24 for those initially randomized to the risdiplam treatment arm. The external comparator population comprised 81 patients from the NatHis-SMA study (NCT02391831) [20, 21] and 57 patients from the placebo arm of a Phase 2 trial of olesoxime (NCT01302600) [22] that both reported MFM scores to match the baseline characteristics of the treated group. The 81 patients from the NatHis-SMA study were aged 2–30 years and included 53 patients with type 2 SMA and 28 patients with ambulant (*n* = 19) or non-ambulant (*n* = 9) type 3 SMA. The 57 patients from the placebo arm of the Phase 2 olesoxime trial were aged 3–25 years and included 39 patients with type 2 SMA and 18 patients with non-ambulant type 3 SMA.

For the external comparator analysis, MFM total score was used for all analyses to compare motor function at month 24. To calculate MFM total score (hereinafter referred to as MFM-derived total score), the external control data were compared with SUNFISH Part 2 data based on the 20-item MFM (MFM20) total score [23] for all patients aged < 6 years and MFM32 total score for all patients aged ≥ 6 years. Both scales were transformed to 0–100%. For the calculation of total domain scores (D1, D2, and D3), within each domain, total domain scores were only calculated if ≤ 15% of items were missing. MFM total scores were only calculated where there was a calculated score for each domain (D1, D2, and D3). Missing MFM total scores were not imputed.

Since the SUNFISH Part 2 study population consisted of non-ambulant patients with types 2 and 3 SMA, ambulant patients were not included in the external control comparison analysis. To ensure robust analysis, patients from the external comparator data set were selected based upon similarities to the SUNFISH Part 2 risdiplam arm patients in terms of demographics, the MFM version, and disease characteristics. After applying the missing item rule on the MFM scale and trimming to exclude patients with missing information on selected prognostic factors (age, SMA type, *SMN2* copy number, presence of scoliosis), 115 patients from the risdiplam arm of SUNFISH Part 2 and 98 patients from the external comparator group who had a valid MFM total score both at baseline and at month 12 or month 24 were included in this analysis.

Patients in the external comparator group were weighted using inverse probability of treatment weighting based upon the selected prognostic factors at baseline, creating a pseudo-population with similar covariate distributions in the treated and untreated groups. A propensity score for each patient was estimated using logistic regression incorporating potential predictors of treatment assignment (risdiplam versus no risdiplam) as independent variables. The potential predictors included in the model were age at baseline (years), SMA type (type 2 or non-ambulant type 3), baseline MFM total score, presence of scoliosis at baseline (yes or no), *SMN2* copy number (2, 3, or 4), and the MFM scale used in the analysis (MFM32 for patients aged ≥ 6 years and MFM20 for those aged < 6 years). Trimming was applied prior to weighting to include only patients with an overlapping distribution of propensity scores. Inverse probability of treatment weighting was applied to the propensity scores to derive weights only in the external comparator group based on the average effect for treated patients, and each patient in the SUNFISH Part 2 risdiplam arm was given a weight of 1.0. To ensure that patients with very low propensity scores would not disproportionately influence the results, weights were truncated at the 99th percentile. In the external comparator population, the weights were summed to generate 114.1 and the sum of weights from patients in SUNFISH Part 2 was 115.0.

Change from baseline in MFM-derived total score was analyzed as an independent variable using a mixed model for repeated measures (MMRM). The independent variables in the MMRM included baseline MFM-derived total score, treatment, time of assessment (i.e., the categorical study visit weeks 17, 26, 35, 52, 78, and 104), treatment-by-time interaction, baseline-by-time interaction, age at baseline, SMA type, *SMN2* copy number, MFM scale used, and presence of scoliosis at baseline. Estimated treatment differences in least-squares mean change from baseline between the risdiplam group and the external comparator group are presented with corresponding 95% confidence intervals (CIs) and *p* values. The proportion of patients demonstrating a change of ≥ 0 and a change of ≥ 3 in MFM total score were analyzed via logistic regression.

## Results

### Patients

A total of 180 patients were enrolled in SUNFISH Part 2 and were randomized to receive risdiplam (*n* = 120) or placebo (*n* = 60) for 12 months (Fig. S1 of Online Resource). Four patients discontinued the study during the placebo-controlled period (risdiplam: *n* = 3, placebo: *n* = 1) to start a commercially available treatment [13]. The discontinued patient randomized to placebo never received risdiplam, and so was excluded from the month 24 exploratory efficacy analyses.

After month 12, all patients received risdiplam. A total of 176 patients entered the open-label treatment period (defined as months 12–24 in the study). Motor function scores from the 59 patients who switched from placebo to risdiplam at study month 12 (i.e., at adjusted baseline) are presented in Table S1. Patients and all individuals in direct contact with patients at the site remained blinded to the treatment group from randomization until completion of at least their second year in the study.

During the open-label treatment period, two patients withdrew from treatment. One of these patients elected to withdraw from the study but completed the month 24 study visit. The other patient withdrew prematurely due to the COVID-19 pandemic and did not complete the month 24 visit. Data are available from 164 patients who were recorded as having completed the open-label treatment period at month 24 by the clinical cut-off date of 30th September 2020 (10 patients did not have a recorded date of completion for the open-label treatment period). The clinical cut-off date is the date at which it was estimated that the last patient in Part 2 would have completed the month 24 study visit. However, some patients missed this study visit due to COVID-19 pandemic restrictions. All patients remaining in the study entered the open‑label extension phase (≥ 24 months) for 3 years of further treatment.

### Motor function

#### MFM32

**Fig. 1:**
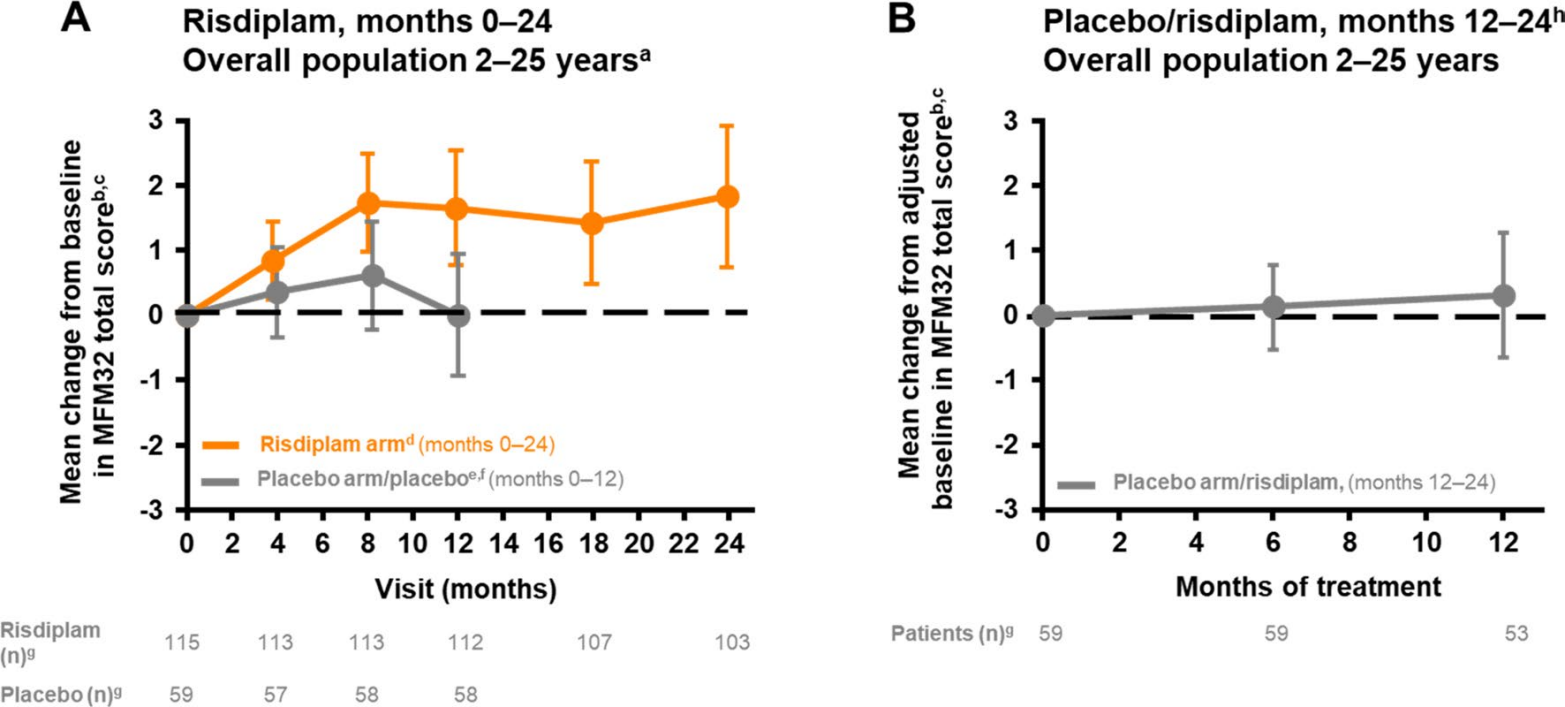
Change from baseline in MFM32 total score in patients treated with risdiplam for up to 24 months and those who previously received placebo until study month 12. ^a^Thirty-one percent (55/180) of the SUNFISH intent-to-treat population were 2–5 years old at baseline. ^b^± 95% CI. ^c^Baseline is the last measurement prior to the first dose of risdiplam or placebo. ^d^Data cut-off: 30 Sep 2020. ^e^Data cut-off: 6 Sep 2019. ^f^Patients in the placebo arm received placebo for 12 months followed by risdiplam treatment for 12 months. ^g^Number of patients with valid results = number of patients with an available total score (result) at respective time points. Intent-to-treat patients. ^h^Patients in the placebo arm received placebo for 12 months followed by risdiplam treatment for 12 months. Placebo period not shown in this graph. *CI* confidence interval, *MFM32* 32-item motor function measure

In patients who initially received risdiplam, the mean change from baseline in MFM32 total score at month 24 was 1.8 (95% CI 0.7–2.9) (Table 1, Fig. 1A). Overall, 58% of patients experienced stabilization in their MFM32 total score (a change of ≥ 0) and 32% achieved an improvement of ≥ 3 in MFM32 total score after 24 months of risdiplam treatment (Table 1).

**Table 1:** Exploratory efficacy endpoints at months 12, 18, and 24

Exploratory efficacy endpoint	Risdiplam group (95% CI) month 12	Risdiplam group (95% CI) month 18	Risdiplam group (95% CI) month 24	Placebo group switched to risdiplam (95% CI) month 12 on risdiplam
Motor function				
Mean change from baseline in MFM32 total score	1.7 (0.8–2.5)^a^	1.4 (0.5–2.4)^b^	1.8 (0.7–2.9)^c^	0.3 (– 0.7 to 1.3)^d^
Proportion of patients with a change of ≥ 3 points in MFM32 total score, %	38% (29.4–47.8)^a^	32% (23.8–41.5)^b^	32% (23.8–41.5)^c^	16% (7.4–27.4)^d^
Proportion of patients with a change of ≥ 0 points in MFM32 total score, %	70% (60.3–77.8)^a^	65% (55.8–73.9)^b^	58% (48.7–67.4)^c^	59% (44.9–71.4)^d^
LS mean change from baseline in MFM32 domain score				
D1 (standing and transfers)	0.4 (– 0.2 to 0.9)^e^	0.5 (0.1–0.9)^f^	0.4 (– 0.1 to 1.0)^g^	0.1 (– 0.7 to 0.8)^h^
D2 (axial and proximal motor function)	1.6 (0.0–3.2)^i^	0.4 (– 1.4 to 2.1)^j^	1.1 (– 0.8 to 3.0)^g^	– 0.3 (– 2.2 to 1.5)^h^
D3 (distal motor function)	4.1 (2.5–5.8)^a^	5.4 (3.7–7.1)^k^	6.3 (4.2–8.3)^c^	2.0 (0.4–3.5)^h^
D1 + D2	1.0 (0.1–1.8)^i^	0.4 (– 0.5 to 1.3)^f^	0.8 (– 0.3 to 1.8)^g^	– 0.1 (– 1.2 to 0.9)^h^
D2 + D3	2.5 (1.1–3.9)^a^	2.1 (0.5–3.6)^k^	2.8 (1.1–4.5)^c^	0.5 (– 1.0 to 2.0)^d^
Mean change from baseline in RULM total score	1.9 (1.2–2.6)^l^	2.1 (1.3–2.8)^k^	2.8 (1.9–3.6)^g^	0.9 (0.1–1.6)^m^
Proportion of patients with a change of ≥ 2 points in RULM total score, %	50% (40.3–58.9)^l^	50% (41.1–59.7)^k^	52% (42.8–61.3)^g^	34% (22.1–47.4)^m^
Mean change from baseline in HFMSE total score	1.2 (0.5–1.9)^n^	1.4 (0.7–2.2)^o^	2.2 (1.1–3.2)^p^	0.0 (– 1.0 to 1.1)^q^
Proportion of patients with a change of ≥ 2 points in HFMSE total score, %	39% (30.4–48.5)^n^	39% (30.4–48.5)^o^	45% (35.9–54.4)^p^	24% (13.6–36.6)^q^
Respiratory				
In all patients				
Mean change from baseline in best percentage-predicted SNIP (%)	4.0 (0.7–7.3)^i^	2.3 (– 1.1 to 5.7)^p^	3.4 (– 1.0 to 7.8)^r^	0.6 (– 3.6 to 4.8)^s^
In patients aged (at screening) 6–25 years only				
Mean change from baseline in best percentage-predicted FVC (%)	– 5.2 (–7.8 to –2.6)^r^	– 6.4 (– 9.5 to – 3.2)^t^	– 7.8 (– 11.6 to – 3.9)^u^	– 3.4 (– 8.0 to 1.3)^v^
Mean change from baseline in best percentage-predicted FEV1 (%)	– 3.8 (– 7.2 to – 0.4)^r^	– 4.7 (– 8.4 to – 1.1)^t^	– 6.9 (– 11.2 to – 2.6)^u^	– 4.1 (– 9.0 to 0.7)^v^
Mean change from baseline in best percentage-predicted MIP (%)	1.0 (– 7.5 to 9.4)^w^	3.7 (–3.7 to 11.2)^x^	5.4 (–3.2 to 14.1)^y^	– 5.7 (– 15.9 to 4.5)^v^
Mean change from baseline in best percentage-predicted MEP (%)	– 2.5 (– 6.1 to 1.1)^r^	– 4.0 (– 8.3 to 0.3)^z^	0.5 (– 5.9 to 6.9)^aa^	– 0.8 (– 5.4 to 3.7)^bb^
Mean change from baseline in best percentage-predicted PCF (%)	0.8 (– 1.4 to 3.0)^r^	– 0.5 (– 2.9 to 1.9)^t^	0.3 (– 2.7 to 3.4)^aa^	0.5 (– 2.7 to 3.6)^v^
Patient-/caregiver-reported outcomes				
In all patients				
Mean change from baseline in the caregiver-reported SMAIS-ULM total score	1.7 (0.8–2.6)^a^	2.1 (1.2–3.1)^k^	2.7 (1.7–3.7)^c^	1.6 (0.4–2.8)^m^
In patients aged 12–25 years only				
Mean change from baseline in the patient-reported SMAIS-ULM total score	1.0 (– 0.2 to 2.1)^cc^	1.0 (– 0.3 to 2.4)^dd^	0.8 (– 0.8 to 2.4)^dd^	0.6 (– 1.0 to 2.2)^ee^

*CI* confidence interval, *FEV1* forced expiratory volume in 1 s, *FVC* forced vital capacity, *HFMSE* Hammersmith Functional Motor Scale—Expanded, *LS* least squares, *MEP* maximal expiratory pressure, *MFM32* 32-item Motor Function Measure, *MIP*, maximal inspiratory pressure, *PCF* peak cough flow, *RULM* revised upper limb module, *SMA* spinal muscular atrophy, *SMAIS-ULM* SMA Independence Scale-Upper Limb Module, *SNIP* sniff nasal inspiratory pressure

Symbols denote n’s: ^a^112, ^b^107, ^c^103, ^d^50, ^e^115, ^f^110, ^g^105, ^h^51, ^i^114, ^j^111, ^k^108, ^l^116, ^m^53, ^n^117, ^o^109, ^p^106, ^q^49, ^r^82, ^s^44, ^t^77, ^u^63, ^v^33, ^w^81, ^×^75, ^y^61, ^z^76, ^aa^62, ^bb^32, ^cc^43, ^dd^39, ^ee^24

Placebo patients switched to risdiplam after 12 months. Baseline is the last measurement prior to the patient’s first dose of risdiplam. Percentages are based on the number of available results at baseline

MFM32 domain scores showed a numerical improvement over 24 months, with a mean change from baseline (95% CI) of 0.4 (– 0.1 to 1.0) in D1 (standing, transfers, and ambulation), 1.1 (– 0.8 to 3.0) in D2 (proximal and axial function), and 6.3 (4.2–8.3) in D3 (distal function) in patients randomized to risdiplam at month 24; gains were also observed for the combined domain scores (D1 + D2 and D2 + D3) (Table 1).

In patients who initially received placebo, the mean change from adjusted baseline (month 12, before the switch to risdiplam) in MFM32 total score was 0.3 (– 0.7 to 1.3) after 12 months of risdiplam treatment (Table 1, Fig. 1B). Overall, 59% of patients originally assigned to placebo experienced stabilization in MFM32 total score (a change of ≥ 0) after 12 months of risdiplam treatment, and 16% demonstrated an improvement (a change of ≥ 3; Table 1). Mean change from baseline (95% CI) in MFM32 domain scores in these patients was 0.1 (– 0.7 to 0.8) for D1, – 0.3 (– 2.2 to 1.5) for D2, and 2.0 (0.4–3.5) for D3 at month 24 (Table 1).

#### MFM-derived total score: SUNFISH (month 24) versus external comparator

**Fig. 2:**
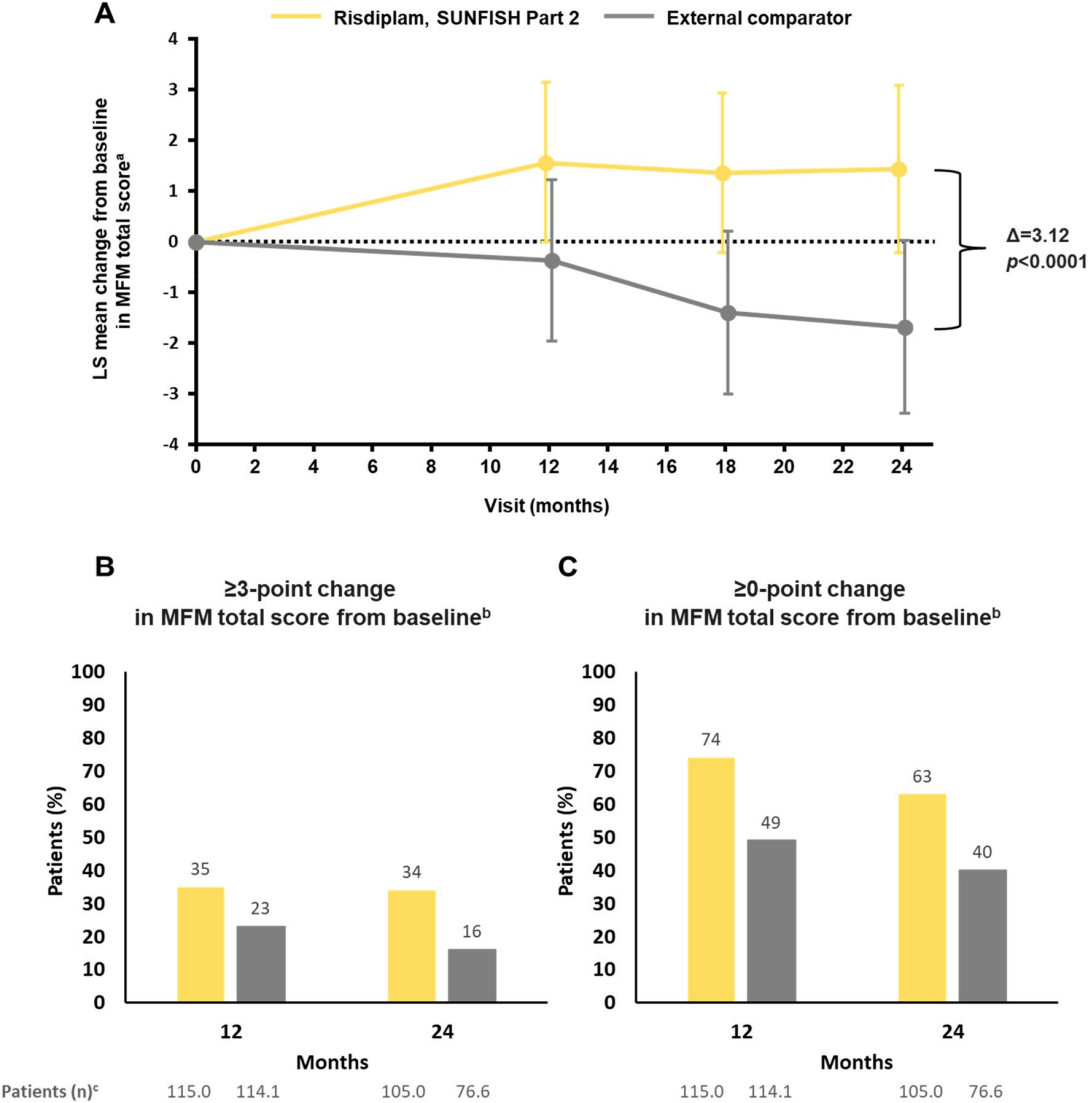
MFM-derived total score in the risdiplam group versus the external comparator group. ^a^± 95% CI, weighted analysis of change from baseline, MMRM (risdiplam, SUNFISH Part 2 *n* = 115.0; external comparator *n* = 114.1, *n* = sum of weights at baseline). Patients with baseline and at least one post-baseline time point at month 12 or month 24 with MFM (derived) total score were included in the analysis. MFM (derived) total score means the MFM20 total score is used for all patients aged < 6 years and MFM32 total score is used for all patients aged ≥ 6 years. Both scales were transformed to 0–100%. SUNFISH data cut-off: 30 Sep 2020. ^b^Weighted analysis. n = sum of weights. ^c^SUNFISH data cut-off: 30 Sep 2020. *CI* confidence interval, *LS* least squares, *MFM* Motor Function Measure, *MFM20* 20-item MFM, *MFM32* 32-item MFM, *MMRM* mixed model for repeated measures, *SD* standard deviation

Baseline characteristics were similar across the risdiplam and the external comparator group (Table 2). Results at months 12 and 24 are shown in Table 3 and Fig. 2. At month 24, the least-squares mean change from baseline (95% CI) was 1.4 (– 0.2 to 3.1) in the risdiplam group and – 1.7 (– 3.4 to 0.0) in the external comparator group, resulting in a least-squares mean treatment difference of 3.1 (1.7–4.6, *p* < 0.0001) (Table 3, Fig. 2). Sixty-three percent of the SUNFISH Part 2 group demonstrated stabilization in MFM total score (a change of ≥ 0 points) at month 24 versus 40% of the external comparator group (odds ratio [OR] 2.7, 95% CI 1.4–5.1), and 34% of the SUNFISH Part 2 group demonstrated an improvement (a change of ≥ 3 points) versus 16% of the external comparator group (OR 2.5, 95% CI 1.1–5.6).

**Table 2:** Baseline patient characteristics in the risdiplam and external comparator groups

	SUNFISH Part 2 risdiplam arm, patients after weighting (*n* = 115.0)^a^	External comparator (*n* = 114.1)^a^
Age at enrollment, years, median (range)	10 (2–25)	8 (2–28)
Age group		
2–5	34.0 (30)	33.2 (29)
6–11	37.0 (32)	35.9 (31)
12–18	34.0 (30)	32.5 (29)
> 18	10.0 (9)	12.5 (11)
Gender, *n* (%)		
Female	60.0 (52)	62 (54)
Male	55.0 (48)	52 (46)
SMA type, *n* (%)		
2	81.0 (70)	81.5 (71)
3	34.0 (30)	32.6 (29)
*SMN2* copy number, *n* (%)		
2	3.0 (2.6)	2.6 (2.3)
3	103.0 (89.6)	102.2 (89.6)
4	9.0 (7.8)	9.2 (8.1)
Scoliosis, *n* (%)	75.0 (65)	74.8 (66)
MFM total score, mean (SD)^b^		
MFM20	(*n* = 34.0)	(*n* = 33.2)
	51.1 (10.7)	49.1 (12.6)
MFM32	(*n* = 81.0)	(*n* = 80.9)
	45.5 (12.7)	46.2 (13.0)

*MFM* Motor Function Measure, *MFM20* 20-item MFM, *MFM32* 32-item MFM,* SD* standard deviation, *SMA* spinal muscular atrophy, *SMN* survival of motor neuron

^a^*n* = sum of weights

^b^MFM (derived) total score means the MFM20 total score is used for all patients aged < 6 years and the MFM32 total score is used for all patients aged ≥ 6 years. Both scales were transformed to 0–100%.

SUNFISH data cut-off: 30 Sep 2020

**Table 3:** Analyses results of MFM-derived total score in the risdiplam group versus the external comparator group

MFM-derived total score^a^	Risdiplam (weighted *n* = 115.0)^b^	External comparator (weighted *n* = 114.1)^b^
Month 24		
LS mean change from baseline at month 24,		–1.7 (–3.4 to 0.0)
Mean (95% CI)	1.4 (–0.2 to 3.1)	
Difference from external comparator,		
LS mean (95% CI)^c^ *p* value	﻿3.1 (1.7–4.6) *p* < 0.0001	
Proportion of patients with a change of ≥ 3 points	34%	16%
OR (95% CI) *p* value	2.5 (1.1–5.6) *p* = 0.0253	
Proportion of patients with a change of ≥ 0 points	63%	40%
OR (95% CI) *p* value	2.7 (1.4–5.1) *p* = 0.0029	

MFM-derived total score^a^	Risdiplam (weighted *n* = 115.0)^a^	External comparator (weighted *n* = 114.1)^a^
Month 12		
Baseline, mean total score (SD)	47.2 (12.3)	47.1 (12.9)
LS mean change from baseline at month 12,		–0.4 (–2.0 to 1.2)
Mean (95% CI)	1.6 (–0.0 to 3.1)	
Difference from external comparator,		
LS mean (95% CI)^c^ *p* value	1.9 (0.7–3.2) *p* = 0.002	
Proportion of patients with a change of ≥ 3 points	35%	23%
Proportion of patients with a change of ≥ 0 points	74%	49%

*CI* confidence interval, *LS* least squares, *MFM* Motor Function Measure*, MFM20* 20-item MFM*, MFM32* 32-item MFM*, MMRM* mixed model for repeated measures, *OR* odds ratio, *SD* standard deviation

^a^MFM (derived) total score means the MFM20 total score is used for all patients aged < 6 years and the MFM32 total score is used for all patients aged ≥ 6 years. Both scales were transformed to 0–100% [37]. SUNFISH data cut-off: 30 Sep 2020

^b^Sum of weights at baseline

^c^MMRM analysis. Weighted analysis of change from baseline. For the analysis, patients with a baseline result and at least one post-baseline result at month 12 or month 24 are included in the analysis. SUNFISH data cut-off: 30 Sep 2020

#### RULM

**Fig. 3:**
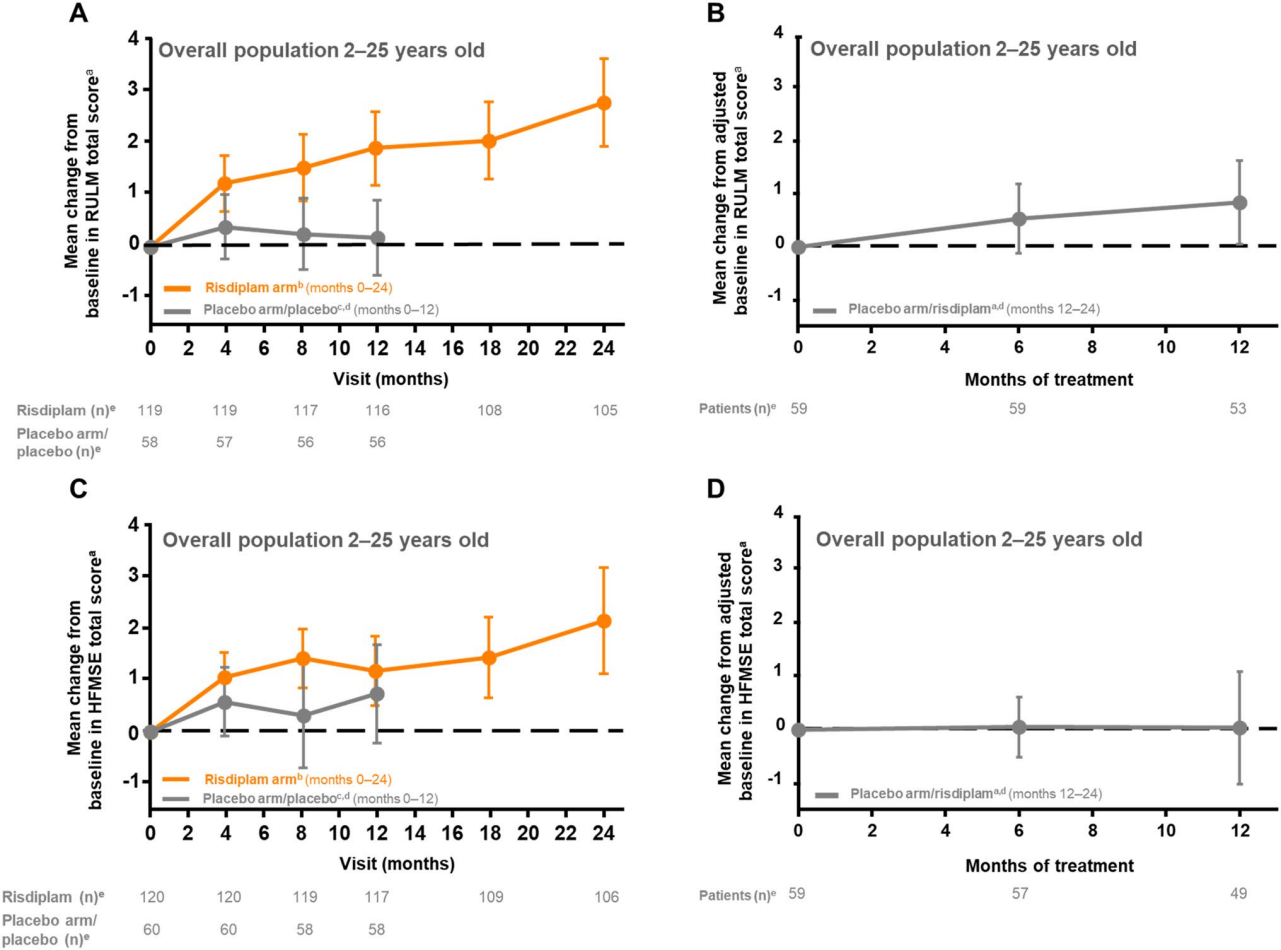
Change in RULM and HFMSE total score from baseline in patients receiving risdiplam for up to 24 months and those who previously received placebo up to study month 12. ^a^± 95% CI. ^b^Data cut-off: 30 Sep 2020. ^c^Data cut-off: 6 Sep 2019. ^d^Patients in the placebo arm received placebo for 12 months followed by risdiplam treatment for 12 months. ^e^Number of patients with valid results = number of patients with an available total score (result) at respective time points. Intent-to-treat patients. *CI* confidence interval, *HFMSE* Hammersmith Functional Motor Scale—Expanded, *RULM* Revised Upper Limb Module

At month 24, the mean change from baseline (95% CI) in RULM total score was 2.8 (1.9–3.6) (Table 1, Fig. 3A); 52% of patients achieved an improvement of ≥ 2 (Table 1). In patients who initially received placebo, the mean change from baseline in RULM total score was 0.9 (0.1–1.6) after 12 months of risdiplam treatment (Table 1, Fig. 3B), and 34% of patients exhibited a change from baseline in RULM total score of ≥ 2 (Table 1).

#### HFMSE

At month 24, the mean change from baseline (95% CI) in HFMSE total score was 2.2 (1.1–3.2) (Table 1, Fig. 3C). Overall, 45% of patients achieved an improvement of ≥ 2 in HFMSE score after 24 months of risdiplam treatment (Table 1). In patients who initially received placebo, the mean change from baseline in HFMSE total score was 0.0 (– 1.0 to 1.1) after 12 months of risdiplam treatment (Table 1, Fig. 3D) and 24% of patients exhibited a change from baseline in HFMSE total score of ≥ 2 (Table 1).

### Respiratory function

#### FVC

FVC was assessed in patients aged 6–25 years at screening. In patients who received risdiplam for 24 months, the mean change from baseline in the best percentage-predicted FVC (95% CI) was – 7.8% (– 11.6 to – 3.9%) after 24 months of risdiplam treatment (Table 1). In patients who initially received placebo, the mean change from baseline in FVC was – 3.4% (– 8.0 to 1.3%) after 12 months of risdiplam treatment (Table 1).

### Caregiver- and patient-reported independence

#### SMAIS-ULM

**Fig. 4:**
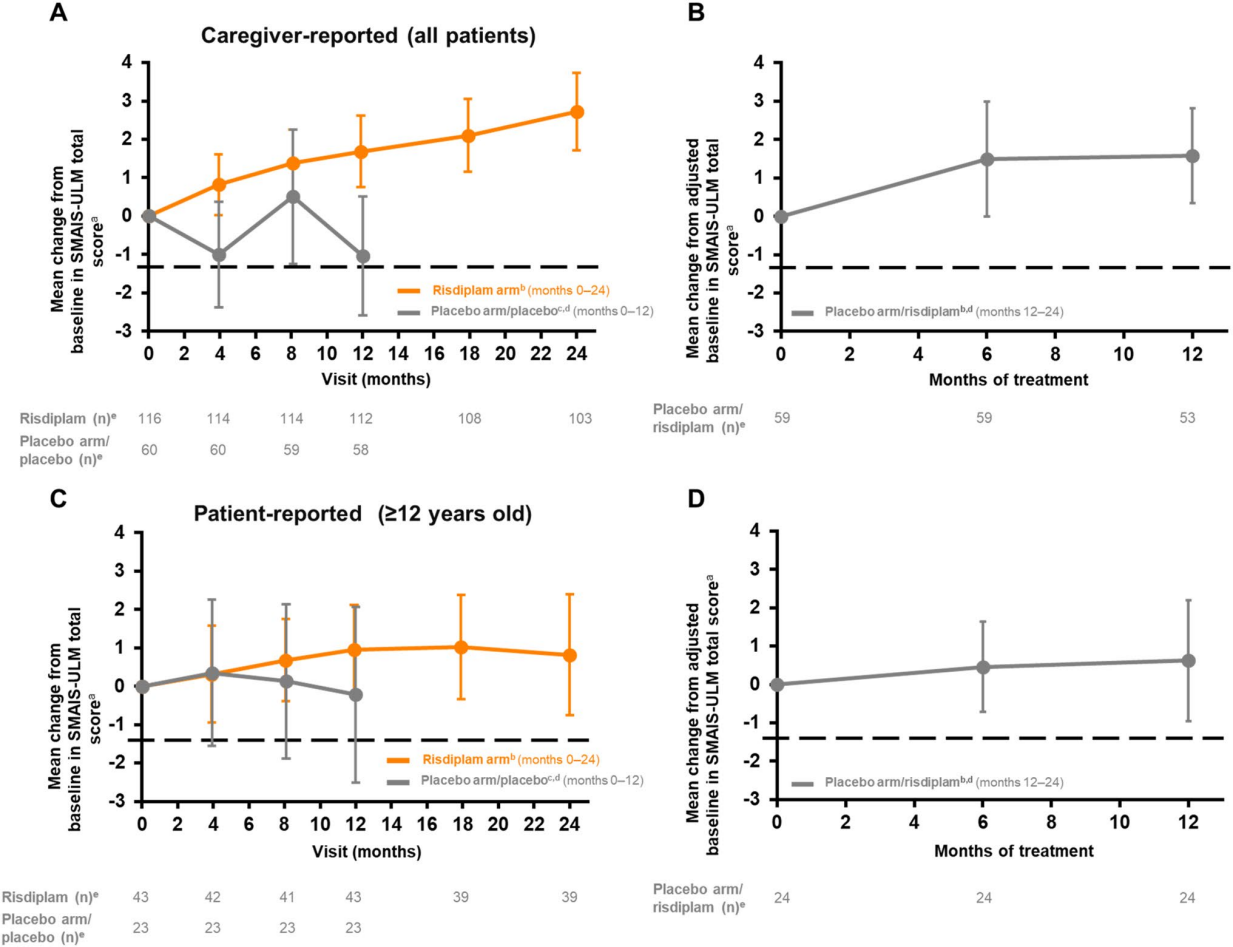
Change in caregiver- and patient-reported SMAIS upper limb total score from baseline in patients receiving risdiplam for up to 24 months and those who previously received placebo up to study month 12. ^a^± 95% CI. Baseline is the last measurement prior to the first dose of risdiplam or placebo. ^b^Data cut-off: 30 Sep 2020. ^c^Data cut-off: 6 Sep 2019. ^d^Patients in the placebo arm received placebo for 12 months followed by risdiplam treatment for 12 months. Risdiplam period not shown in this graph. ^e^Number of patients with valid results = number of patients with an available total score (result) at respective time points. Intent-to-treat patients. SMAIS scores range from 0 to 44 following rescoring to a 0–2 response scale for each item. Higher scores indicate greater independence in completing daily activities. *CI* confidence interval, *SMA* spinal muscular atrophy, *SMAIS* SMA Independence Scale, *SMAIS-ULM* SMA Independence Scale-Upper Limb Module

In patients who received risdiplam for 24 months, the mean change from baseline (95% CI) in the caregiver-reported SMAIS-ULM total score was 2.7 (1.7–3.7) at month 24 (Table 1, Fig. 4A); the mean change from baseline in the patient-reported SMAIS-ULM total score (patients aged 12–25 years only) was 0.8 (– 0.8 to 2.4) (Table 1, Fig. 4C).

In patients who initially received placebo, the mean change from baseline in the caregiver-reported SMAIS-ULM total score was 1.6 (0.4–2.8) after 12 months of risdiplam treatment (Table 1, Fig. 4B); the mean change from baseline in patient-reported SMAIS-ULM total score was 0.6 (– 1.0 to 2.2) (Table 1, Fig. 4D).

### Safety results

Safety results in all patients enrolled in SUNFISH Part 2 (*n* = 180) are summarized in Table 4. A total of 91.7% of patients in the risdiplam arm experienced at least one AE between months 12 and 24. In patients initially treated with placebo, 91.7% experienced at least one AE between months 0 and 12 while receiving placebo. After switching to risdiplam at month 12, 80.0% of patients experienced at least one AE between months 12 and 24. The most commonly reported AEs were upper respiratory tract infection, nasopharyngitis, pyrexia, headache, diarrhea, vomiting, and cough.

**Table 4:** Safety summary

		Risdiplam arm 0–12 months (*n* = 120)^a^ [13]	Risdiplam arm 12–24 months (*n* = 120)^b^	Placebo arm/placebo 0–12 months (*n* = 60)^a^ [13]	Placebo arm/risdiplam 12–24 months (*n* = 60)^b^
Patients with at least one AE, *n* (%)		111 (92.5)	110 (91.7)	55 (91.7)	48 (80.0)
Total number of AEs		789	506	354	242
Total number of deaths		0	0	0	0
Total number of patients with at least one, *n* (%)					
AE with fatal outcome		0	0	0	0
SAE		24 (20.0)	25 (20.8)	11 (18.3)	3 (5.0)
SAE leading to withdrawal from treatment		0	0	0	0
SAE leading to dose modification/interruption		4 (3.3)	4 (3.3)	2 (3.3)	0
Treatment-related SAE		0	0	0	0
AE leading to withdrawal from treatment		0	0	0	0
AE leading to dose modification/interruption		8 (6.7)	7 (5.8)	2 (3.3)	2 (3.3)
Treatment-related AE		16 (13.3)	5 (4.2)	6 (10.0)	3 (5.0)
Related AE leading to dose modification/interruption		0	0	0	0
Grade 3–5 AE		21 (17.5)	19 (15.8)	8 (13.3)	3 (5.0)
Most common AEs, *n* (number of patients [%])					
Upper respiratory tract infection		38 (31.7)	19 (15.8)	18 (30.0)	10 (16.7)
Nasopharyngitis		31 (25.8)	26 (21.7)	15 (25.0)	6 (10.0)
Pyrexia		25 (20.8)	16 (13.3)	10 (16.7)	6 (10.0)
Headache		24 (20.0)	12 (10.0)	10 (16.7)	10 (16.7)
Diarrhea		20 (16.7)	9 (7.5)	5 (8.3)	6 (10.0)
Vomiting		17 (14.2)	14 (11.7)	14 (23.3)	8 (13.3)
Cough		17 (14.2)	12 (10.0)	12 (20.0)	5 (8.3)
Most common SAEs, *n* (number of patients [%])					
Pneumonia		9 (7.5)	8 (6.7)	1 (1.7)	0 (0)

*AE* adverse event, *SAE* serious AE

^a^Month 12 data cut-off: 6 Sep 2019

^b^Month 24 data cut-off: 30 Sep 2020

The most commonly reported SAE was pneumonia. In the risdiplam arm, 20.0% of patients experienced at least one SAE between months 0 and 12, and 20.8% experienced at least one SAE between months 12 and 24. In patients initially receiving placebo, 18.3% experienced at least one SAE between months 0 and 12 when receiving placebo. After switching to risdiplam at month 12, 5.0% experienced at least one SAE between months 12 and 24. In the 179 patients exposed to risdiplam, the rate of overall SAEs per 100 patient-years (PY; 95% CI) remained relatively consistent over time, with 26.7 AEs/100 PY (17.1–39.7) during the 0 to ≤ 6 months period, 19.4 AEs/100 PY (11.3–31.1) during the > 6 to ≤ 12 months period, 36.1 AEs/100 PY (23.6–52.8) during the > 12 to ≤ 18 months period, and 26.8 AEs/100 PY (15.3–43.4) during the > 18 to ≤ 24 months period.

In patients who received risdiplam for 24 months, Grade 3–5 AEs were reported in 17.5% of patients between months 0 and 12, and in 15.8% of patients between months 12 and 24. In patients who initially received placebo, Grade 3–5 AEs were reported in 13.3% of patients between months 0 and 12. After switching to risdiplam at month 12, 5.0% of patients experienced at least one Grade 3–5 AE. No Grade 5 (fatal) AEs were reported. No SAEs were reported as being related to risdiplam treatment. There were no AEs or SAEs leading to withdrawal from treatment.

## Discussion

The natural history of types 2 and 3 SMA involves progression of disease and continued loss of function [21, 24]. However, disease trajectories differ according to age and disease severity. Published natural history studies in untreated patients with types 2 and 3 SMA (ambulant and non-ambulant) have reported decline in RULM total score over 12 months (– 0.4 points in patients aged 2.7–49.7 years) [25] and 24 months (– 0.79 points in patients aged 5–56 years) [26] and HFMSE total score over 12 months (– 0.54 points in children and adults aged ≥ 2 years with a diagnosis before 19 years of age) [24], and significant decline over 24 months in MFM32 total score (– 2.08 points in patients aged 2–30 years) [21]. Although a 2-point (RULM [27]) or 3-point (HFMSE [27], MFM32 [28]) change in these functional motor scales has been highlighted as a clinically meaningful change, stabilization in motor function is an important goal identified by patients with types 2 and 3 SMA [29, 30] and is, thus, considered a clinically meaningful outcome in this population.

### Motor function and independence

Risdiplam treatment in a clinically heterogeneous population of children, teenagers, and adults with later-onset SMA and varying disease duration resulted in continued stabilization or improvement in motor function. Although analyses are exploratory, overall, the gains in motor function after 12 months of risdiplam treatment were maintained or improved upon up to month 24—confirming the benefit of longer-term risdiplam treatment.

In patients with prolonged disease duration, the improvement with treatment is not expected to be evident in the short term, as it requires the activation of multiple compensatory reinnervation processes. Furthermore, hip contractures in non-ambulant patients with prolonged disease duration limit functional gains in tasks that require full hip extension. Thus, a goal of treatment is the long-term stability of specific functions, many of which involve the hands or upper extremities, which are important for the autonomy of non-ambulant patients. The progressive improvement on the RULM scale (1.91 at month 12, 2.06 at month 18, and 2.79 at month 24) following risdiplam treatment in this study exemplifies the impact of risdiplam on upper limb function. Increases in the caregiver-reported SMAIS-ULM total score (1.68 at month 12, 2.10 at month 18, and 2.73 at month 24; available for the full population), which is strongly correlated with the RULM [19], also corroborate the consistent effect of risdiplam on upper limb function in this population. Taken alongside numerical increases in the HFMSE total score and the patient-reported SMAIS-ULM that were also observed at month 24, this provides evidence that risdiplam is providing benefit to treated patients relative to untreated control patients.

The trajectories of MFM32 and RULM total scores changed in patients who switched from placebo to risdiplam at month 12, while decline was observed from baseline to month 12 (when patients received placebo). A trend toward improvement or stabilization in motor function was observed from months 12 to 24 (12 months after the switch to risdiplam treatment).

The external comparator analysis showed that risdiplam administration led to significant improvements in motor function at months 12 and 24 (Table 3, Fig. 2). The change from baseline over 12 months in the comparator analysis is consistent with the treatment difference between risdiplam and placebo in the primary analysis of SUNFISH Part 2 at month 12, which reported a mean treatment difference (95% CI) of 1.55 (0.30–2.81, *p* = 0.016) in MFM32 total score in favor of risdiplam [13].

In the comparison, analyses of MFM-derived total scores showed that risdiplam treatment in SUNFISH Part 2 led to an increase in mean score from baseline to month 24, which was significantly different from the progressive decline observed in the untreated external comparator. After 24 months of treatment, a higher proportion of individuals treated with risdiplam showed improvement or stabilization (≥ 3- or ≥ 0-point change, respectively) in MFM total score compared with the untreated external comparator. These results provide further confirmation of the longer-term efficacy of risdiplam in a broad population of patients with type 2 and non-ambulant type 3 SMA compared with untreated patients.

Importantly, although risdiplam increases levels of functional SMN protein [31], this may not restore lost motor neurons [9]. Modest declines in compound muscle action potential of − 0.007 mV per month can be seen over even short timeframes in patients with type 2 SMA [32]. The dynamics of change in risdiplam-treated patients suggest improvement in motor function, which is more pronounced in younger patients [13], followed by stabilization. A possible explanation for the improvement and subsequent stabilization, as well as the enhanced treatment benefit to younger patients, is that patients with SMA have a limited pool of motor neurons, and these neurons may be in a state of metabolic distress [33, 34]. Treatment enables recruitment of these motor neurons, which improves motor function, but this benefit is still limited by the number of remaining motor neurons. As older patients have fewer remaining motor neurons, their potential for improvement on standardized motor scales is reduced; this may help explain why less improvement was observed in patients who switched from placebo to risdiplam (and were, thus, older when their risdiplam treatment was initiated) relative to patients who initially received risdiplam treatment. In these older patients with more advanced disease, stabilization of motor function is an important goal of treatment.

### Respiratory function

Best percentage-predicted FVC in this population declined at a rate consistent with natural history findings [35], showing a lack of risdiplam-induced improvement in this measure. Similar results were observed in a single-center study of the antisense oligonucleotide nusinersen in 12 patients with types 2 and 3 SMA aged 4–12 years; this study reported no improvement in FVC 300 days after treatment, although best percentage-predicted FVC was not measured [36]. The authors attributed this finding to the late initiation of nusinersen in the disease course and the lack of evidence that nusinersen affects contractures caused by peripheral muscle weakness in SMA (thoracic cage contractures subsequent to muscle weakness are the main cause of thoracic restriction in neuromuscular diseases). The lack of improvement in best percentage-predicted FVC observed in SUNFISH highlights the importance of continuing respiratory standard of care.

### Safety

The safety profile after 24 months of treatment was consistent with the safety results after 12 months of treatment (Table 4). The number of patients who experienced at least one SAE remained stable during the second year (21%) of risdiplam treatment compared with the first year (20%). In contrast, in those patients initially assigned to placebo who switched to risdiplam at month 12, the number of patients reporting at least one SAE decreased during the second year of the study with risdiplam treatment (5%), compared with the first year when they received placebo (18%).

Initial observations in the double-blind period showed a slightly higher incidence of serious pneumonia in patients in the risdiplam arm (8%) compared with the placebo arm (2%). However, the incidence of serious pneumonia did not increase in those patients who switched to risdiplam at month 12 (0%) compared with the first year on placebo (2%). Furthermore, the incidence of pneumonia did not increase during the second year of risdiplam treatment (7%), compared with the first year of risdiplam treatment (8%).

A review of laboratory parameters did not reveal any risdiplam-associated toxicity. There have been no clinically significant safety findings in patients reflective of potential risks previously identified from nonclinical toxicology studies (effects on epithelial tissues, retinal toxicity, or hematologic effects). AEs in the System Organ Class ‘eye disorders’ were not suggestive of risdiplam-induced effects.

## Conclusion

Risdiplam treatment in a broad and clinically heterogeneous patient population of children, teenagers, and adults with later-onset SMA and varying disease duration resulted in continued clinically relevant gains in motor function. This was demonstrated by improvements (32% of patients) and stabilization (58% of patients) in MFM32 total score at month 24 and confirmed by progressive improvements on RULM—an additional, independent measure of upper limb motor function—as well as caregiver-reported SMAIS‑ULM. The gains in motor function observed after 12 months of risdiplam treatment were maintained or improved upon after 24 months, confirming the benefit of longer-term treatment with risdiplam. The safety profile after 24 months of treatment was consistent with that previously observed after 12 months of treatment [13]. The SUNFISH Part 2 24-month data further demonstrate the clinically meaningful benefits of risdiplam and reinforce a positive benefit–risk profile for the treatment of children, teenagers, and adults with later-onset SMA.

## Supplementary Information

Below is the link to the electronic supplementary material.

Supplementary file1 (DOCX 49 kb)

## Data Availability

For eligible studies, qualified researchers may request access to individual patient level clinical data through a data request platform. At the time of writing this request platform is Vivli. https://vivli.org/ourmember/roche/. For up-to-date details on Roche's Global Policy on the Sharing of Clinical Information and how to request access to related clinical study documents, see: https://go.roche.com/data_sharing. Anonymized records for individual patients across more than one data source external to Roche cannot, and should not, be linked due to a potential increase in risk of patient re-identification.
