# Correction to: Two‑year efficacy and safety of risdiplam in patients with type 2 or non‑ambulant type 3 spinal muscular atrophy (SMA)

**DOI:** 10.1007/s00415-023-11658-6

**Published:** 2023-04-18

**Authors:** Maryam Oskoui, John W. Day, Nicolas Deconinck, Elena S. Mazzone, Andres Nascimento, Kayoko Saito, Carole Vuillerot, Giovanni Baranello, Nathalie Goemans, Janbernd Kirschner, Anna Kostera-Pruszczyk, Laurent Servais, Gergely Papp, Ksenija Gorni, Heidemarie Kletzl, Carmen Martin, Tammy McIver, Renata S. Scalco, Hannah Staunton, Wai Yin Yeung, Paulo Fontoura, Eugenio Mercuri, on behalf of The SUNFISH Working Group

**Affiliations:** 1 Departments of Pediatrics and Neurology and Neurosurgery, McGill University, Montreal, Canada; 2 Department of Neurology, Stanford University, Palo Alto, CA USA; 3 Neuromuscular Reference Center, UZ Gent, Ghent, Belgium; 4 Centre de Référence des Maladies Neuromusculaires et Service de Neurologie Pédiatrique, Queen Fabiola Children’s University Hospital, Université Libre de Bruxelles, ULB, Brussels, Belgium; 5 Pediatric Neurology Institute, Catholic University and Nemo Pediatrico, Fondazione Policlinico Gemelli IRCCS, Rome, Italy; 6 Neuromuscular Unit, Neuropaediatrics Department, Hospital Sant Joan de Déu, Fundacion Sant Joan de Deu, CIBERER–ISC III, Barcelona, Spain; 7 Institute of Medical Genetics, Tokyo Women’s Medical University, Tokyo, Japan; 8 Department of Pediatric Physical Medicine and Rehabilitation, Hôpital Mère Enfant, CHU-Lyon, Lyon, France; 9 Neuromyogen Institute, CNRS UMR 5310-INSERM U1217, Université de Lyon, Lyon, France; 10 The Dubowitz Neuromuscular Centre, NIHR Great Ormond Street Hospital Biomedical Research Centre, Great Ormond Street Institute of Child Health, University College London and Great Ormond Street Hospital Trust, London, UK; 11 Developmental Neurology Unit, Fondazione IRCCS Istituto Neurologico Carlo Besta, Milan, Italy; 12 Neuromuscular Reference Centre, Department of Paediatrics and Child Neurology, University Hospitals Leuven, Leuven, Belgium; 13 Department of Neuropediatrics and Muscle Disorders, Faculty of Medicine, Medical Center–University of Freiburg, Freiburg, Germany; 14 Department of Neurology, Medical University of Warsaw, Warsaw, Poland; 15 I-Motion-Hôpital Armand Trousseau, Paris, France; 16 MDUK Oxford Neuromuscular Centre, Department of Paediatrics, University of Oxford, Oxford, UK; 17 Division of Child Neurology, Centre de Références des Maladies Neuromusculaires, University Hospital Liège and University of Liège, Liège, Belgium; 18 Pharma Development, Safety, F. Hoffmann-La Roche Ltd, Basel, Switzerland; 19 PDMA Neuroscience and Rare Disease, F. Hoffmann-La Roche Ltd, Basel, Switzerland; 20 Roche Pharmaceutical Research and Early Development, Roche Innovation Center Basel, Basel, Switzerland; 21 Roche Products Ltd, Welwyn Garden City, UK; 22 Pharma Development Neurology, F. Hoffmann-La Roche Ltd, Basel, Switzerland

**Correction to: Journal of Neurology** 10.1007/s00415-023-11560-1

The original version of this article unfortunately contained a mistake. The corrected details are given below for your reading.

In figure 1, there is an error in the n numbers below the graph in Panel 1b for the placebo group. The n numbers underneath Panel 1b should be 58 58 50.

There is an error within Fig. 4. The dashed lines at ~ − 1.4 should be at 0. They have been moved downwards and are no longer in the correct place.

The corrected Figs. 1 and 4 are given in the following page.

**Fig. 1:**
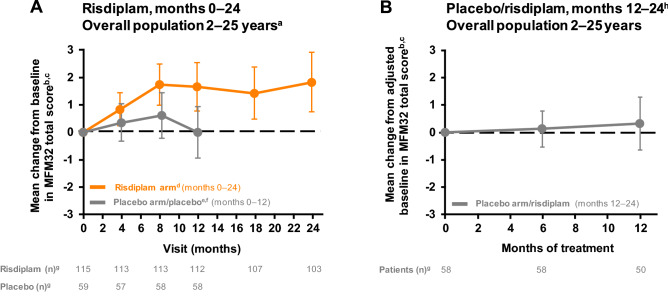
Change from baseline in MFM32 total score in patients treated with risdiplam for up to 24 months and those who previously received placebo until study month 12. ^a^Thirty-one percent (55/180) of the SUNFISH intent-to-treat population were 2–5 years old at baseline. ^b^± 95% CI. ^c^Baseline is the last measurement prior to the first dose of risdiplam or placebo. ^d^Data cut-off: 30 Sep 2020. ^e^Data cut-off: 6 Sep 2019. ^f^Patients in the placebo arm received placebo for 12 months followed by risdiplam treatment for 12 months. ^g^Number of patients with valid results = number of patients with an available total score (result) at respective time points. Intent-to-treat patients. ^h^Patients in the placebo arm received placebo for 12 months followed by risdiplam treatment for 12 months. Placebo period not shown in this graph. *CI* confidence interval, *MFM32* 32-item motor function measure

**Fig. 4:**
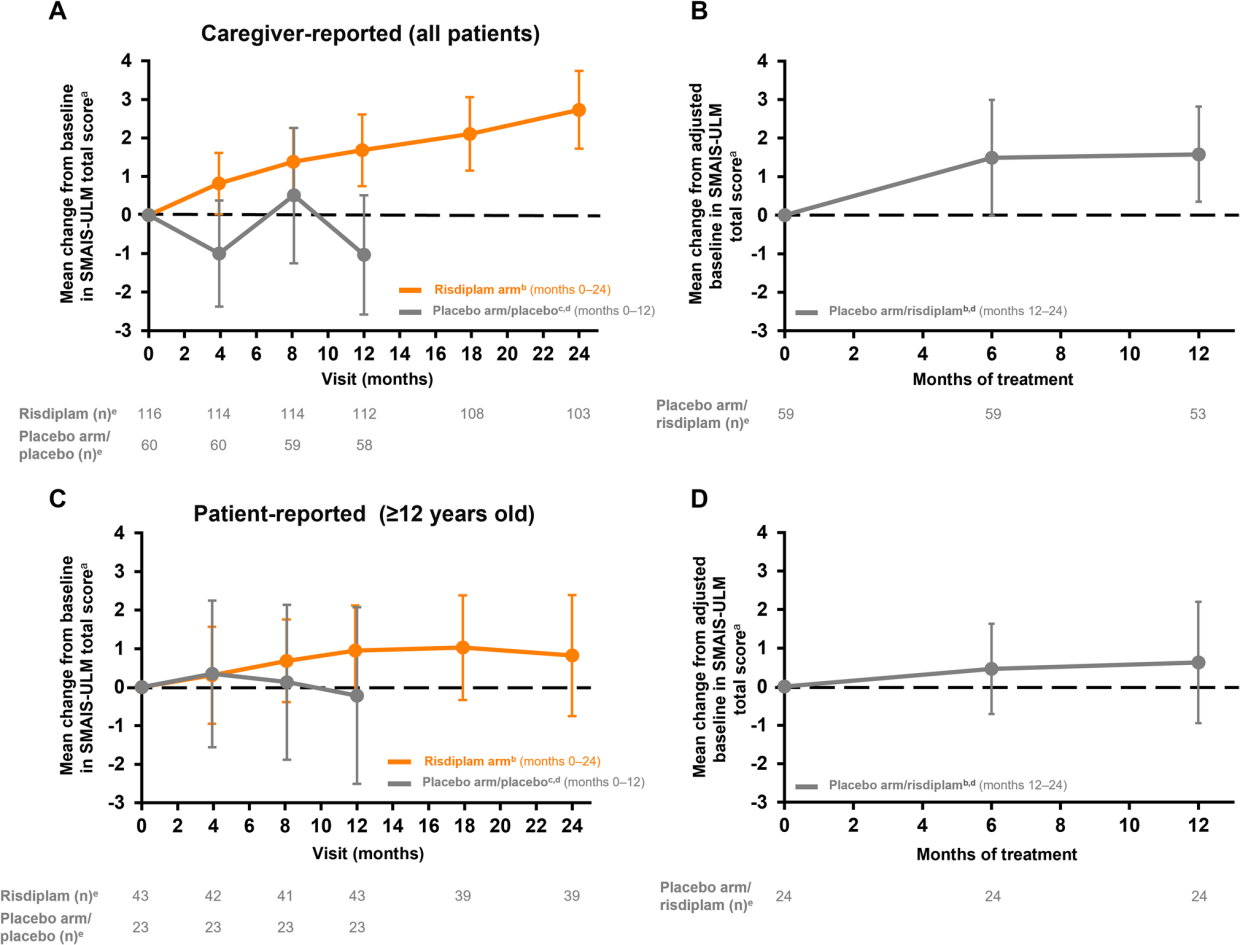
Change in caregiver- and patient-reported SMAIS upper limb total score from baseline in patients receiving risdiplam for up to 24 months and those who previously received placebo up to study month 12. ^a^± 95% CI. Baseline is the last measurement prior to the first dose of risdiplam or placebo. ^b^Data cut-off: 30 Sep 2020. ^c^Data cut-off: 6 Sep 2019. ^d^Patients in the placebo arm received placebo for 12 months followed by risdiplam treatment for 12 months. Risdiplam period not shown in this graph. ^e^Number of patients with valid results = number of patients with an available total score (result) at respective time points. Intent-to-treat patients. SMAIS scores range from 0 to 44 following rescoring to a 0–2 response scale for each item. Higher scores indicate greater independence in completing daily activities. *CI* confidence interval, *SMA* spinal muscular atrophy, *SMAIS* SMA Independence Scale, *SMAIS-ULM* SMA Independence Scale-Upper Limb Module

