# Supplementary material for: Two-year efficacy and safety of risdiplam in patients with type 2 or non-ambulant type 3 spinal muscular atrophy (SMA)
Source: J Neurol. 2023 Feb 3;270(5):2531–46. doi: 10.1007/s00415-023-11560-1 (PMC9897618; doi:10.1007/s00415-023-11560-1)
Supplement: Supplementary file 1 — Supplementary file1 (DOCX 49 kb) [file 415_2023_11560_MOESM1_ESM.docx]

**Figure S1: SUNFISH Part 2 trial profile**

211 patients screened

31 patients excluded^a^

10 did not meet the inclusion criteria

6 did not have a high-quality optical coherence topography

5 met the exclusion criteria

4 withdrew consent

2 could not meet study requirements

2 had an administrative error

1 had an abnormality in laboratory results

1 had planned surgery

180 patients randomized

120 assigned to receive risdiplam

60 assigned to receive placebo

3 discontinued treatment

2 switched to nusinersen

1 switched to unspecified treatment

1 discontinued treatment

1 switched to nusinersen

59 patients completed 12-month placebo‑controlled period and switched to risdiplam

117 patients completed 12-month placebo‑controlled period

176 patients entered the open-label treatment period

2 patients discontinued treatment

1 withdrawal by patient (completed the Week 104 study visit)

1 due to the COVID-19 pandemic (did not complete the Week 104 visit)

10 patients did not have a recorded date of completion for the open-label treatment period

164 patients completed the open-label period and entered open-label extension^a^

^a^The Month 24 visits of 10 patients were delayed due to the COVID-19 pandemic. Data are available from 164 patients who were recorded as having completed the open-label treatment period by the clinical cut-off date of 30th September 2020. The clinical cut-off date is the date at which it was estimated that the last patient in Part 2 would have completed the Week 104 study visit. All 164 patients entered the open-label extension period.

For efficacy analyses for each endpoint, individuals who fulfilled the corresponding missing item rules were excluded as predefined in the statistical analysis plan. Patients who received at least one dose of risdiplam (*n* = 179 patients) were included in the exploratory efficacy analysis. Efficacy data from all timepoints up to Month 24 (or, for those who withdrew early from the study, efficacy data from all timepoints when they were still receiving the treatment) were included in the analyses. All individuals who received at least one dose of risdiplam or placebo were included in the safety analysis (*n* = 120 in the risdiplam group, *n* = 60 in the placebo group).

OCT, optical coherence tomography.

**Table S1: Motor function scores at study Month 12 for patients who switched from placebo to risdiplam (at adjusted baseline, start of risdiplam treatment)**

|  | **Placebo arm/risdiplam**  **Study Month 12** |
| --- | --- |
| MFM32 score, mean (SD)  *n* | 47.14 (10.87)  58 |
| RULM score, mean (SD)  *n* | 20.41 (6.40)  59 |
| HFMSE score, mean (SD)  *n* | 16.76 (11.54)  59 |

*HFMSE* Hammersmith Functional Motor Scale – Expanded, *MFM32* 32-item Motor Function Measure, *RULM* Revised Upper Limb Module.

## SUNFISH Study Group

**Independent Data Monitoring Committee**

Joseph J. Volpe M.D. (Chair), John Posner PhD. (Clinical Pharmacology), Ulrich Kellner M.D. (Ophthalmologist), Rosaline Quinlivan M.D. (Neurologist).

**SUNFISH Principal Investigators (in bold) and site study personnel**

*Belgium (Parts 1 & 2)*: **Aurore Daron M.D.,** Stéphanie Delstanche M.D.; Ophthalmologists: Bruninx Romain; Physiotherapists: Fabian Dal Farra, Olivier Schneider; **Nicolas Deconinck M.D.;** Ophthalmologists: Irina Balikova M.D., Patricia Delbeke M.D., Inge Joniau M.D.; Physiotherapists: Valentine Tahon, Sylvia Wittevrongel; Study Coordinator: Elke De Vos; **Nathalie Goemans M.D.,** Ingele Casteels M.D., Liesbeth De Waele M.D.; Ophthalmologists: Irina Balikova, Catherine Cassiman, Lies Prové; Physiotherapists: David Kinoo, Lisa Vancampenhout, Marleen Van Den Hauwe, Annelies Van Impe; *Brazil (Part 2)*: **Alexandra Prufer de Queiroz Campos Araujo M.D.,** Aline Chacon Pereira M.D., Flávia Nardes M.D.; Ophthalmologists: Lorena Haefeli, Julia Rossetto; Physiotherapists: Jaqueline Almeida Pereira, Marcos Ferreira Rebel; *Canada (Part 2)*: **Craig** **Campbell M.D.;** Ophthalmologist: Sapna Sharan; Physiotherapists: Wendy McDonald, Cheryl Scholtes; **Jean Mah M.D.,** Maria Sframeli M.D.; Physiotherapists: Angela Chiu, Jane Hagel; **Maryam Oskoui M.D.;** Ophthalmologists: Raquel Beneish, Connie Pham, Daniela Toffoli; Physiotherapists: Stephanie Arpin, Sarah Turgeon Desilets; *China (Part 2)*: **Yi Wang M.D.,** Chaoping Hu M.D., Jianfeng Huang M.D; Ophthalmologists: Chen Qian, Li Shen, Ying Xiao, Zhenxuan Zhou; Physiotherapists: Hui Li, Sujuan Wang; **Hui Xiong M.D.,** Xingzhi Chang M.D., Hui Dong M.D., Ying Liu M.D., Tian Sang M.D., Cuijie Wei M.D., Jing Wen M.D.; Ophthalmologists: Yiwen Cao, Xingyao Lv, Jing Wen, Jingjing Zhao; Physiotherapists: Wenzhu Li, Lun Qin; *Croatia*: **Nina Barisic M.D.;** Ophthalmologists: Martina Galiot Delic, Petra Kristina Ivkić, Nenad Vukojević; Physiotherapists: Ivana Kern, Boris Najdanovic, Marin Skugor; *France (Parts 1 & 2)*: **Laurent Servais M.D., Odile Boespflug-Tanguy M.D.,** Andreea Seferian M.D., Claudia Ravelli M.D., Laure Vanden Brande M.D., Ruxandra Cardas M.D., Silvana De Lucia MD, Teresa Gidaro M.D.; Ophthalmologists: Emmanuel Barreau, Nabila Mnafek, Marta Milkova Momtchilova, Helene Peche, Carole Valherie; Physiotherapists: Allison Grange, Charlotte Lilien, Darko Milascevic, Shotaro Tachibana; Pneumologists: Guillaume Aubertin, Jessica Taytard; **Jean-Baptiste Davion M.D.,** Stephanie Coopman M.D.**;** Ophthalmologists: Ikram Bouacha, Philippe Debruyne, Sabine Defoort M.D., Gilles Derlyn, Florian Leroy; Physiotherapists: Loïc Danjoux, Julie Guilbaud; **Isabelle** **Desguerre M.D.,** Christine Barnérias M.D., Michaela Semeraro M.D.; Ophthalmologists: Dominique Bremond-Gignac, Lenaic Bruere, Maxence Rateaux; Physiotherapists: Élodie Deladriere, Virginie Germa; **Yann Pereon M.D.,** Armelle Magot M.D., Sandra Mercier M.D.; Ophthalmologist: Fanny Billaud; Physiotherapists: Lucie Le Goff, Guy Letellier M.D.; **Carole** **Vuillerot M.D.,** Aurélie Portefaix M.D., Camille De-Montferrand M.D., Laure Le-Goff M.D., Stephanie Fontaine M.D.; Ophthalmologist: Nabil Bouzid; Physiotherapists: Aurélie Barriere, Marie Tinat; Study Coordinator: Manel Saidi; *Germany (Part 1)*: **Janbernd Kirschner M.D.;** Ophthalmologists: Michelle Dreesbach, Wolf Lagréze, Bettina Michaelis, Fanni Molnar, Dorina Seger; Physiotherapist: Sibylle Vogt; *Italy (Parts 1 & 2)*: **Enrico** **Bertini M.D.,** Adele D’Amico M.D.; Ophthalmologist: Sergio Petroni; Physiotherapists: Anna Maria Bonetti, Adelina Carlesi, Irene Mizzoni; **Claudio Bruno M.D.;** Ophthalmologists: Enrico Priolo, Giuseppe Rao; Physiotherapists: Simone Morando, Paola Tacchetti, Ambra Zuffi; **Giacomo Pietro Comi M.D.,** Roberta Brusa M.D., Stefania Corti M.D., Velardo Daniele M.D., Alessandra Govoni M.D., Francesca Magri M.D., Megi Meneri M.D.; Ophthalmologists: Valeria Minorini, Silvia Gabriella Osnaghi; Physiotherapists: Francesca Abbati, Federica Fassini, Michaela Foa, Amaqlia Lopopolo; Study Coordinators: Valeria Parente, Francesca Zoppas; **Giovanni Baranello M.D., Riccardo Masson M.D.;** Ophthalmologists: Stefania Bianchi Marzoli, Diletta Santarsiero, Myriam Garcia Sierra, Gemma Tremolada; Physiotherapists: Maria Teresa Arnoldi, Marta Vigano, Riccardo Zanin; **Eugenio Mercuri M.D.;** Laura Antonaci, M.D., Roberto de Sanctis M.D., Marika Pane M.D., Maria Carmela Pera, M.D.; Ophthalmologists: Giulia Maria Amorelli, Costanza Barresi, Gugliemo D’Amico, Lorenzo Orazi; Physiotherapists: Giorgia Coratti; *Japan (Part 2)*: **Kazuhiro Haginoya M.D.;** Physiotherapists: Atsuko Kato, Yuko Morishita; **Ryutaro Kira M.D.;** Ophthalmologists: Kiyomu Akiyama, Miwako Goto, Yujiro Mori, Misato Okamoto, Saki Tsutsui; Physiotherapists: Yuta Takatsuji, Aya Tanaka; **Hirofumi Komaki M.D.;** Physiotherapists: Ippei Suzuki, Mizuki Takeuchi, Daisuke Todoroki; **Seiji Watanabe M.D.;** Ophthalmologists: Miina Omori; **Tomoko Matsubayashi M.D.;** Physiotherapists: Emi Inakazu, Hiroe Nagura, Akira Suzuki; **Hitoshi Osaka M.D.;** Physiotherapists: Manami Ohashi; **Nobutsune Ishikawa M.D.;** Ophthalmologist: Yousuke Harada; Physiotherapists: Kenichi Fudeyasu, Kazuhiko Hirata, Kana Michiue, Kazuyuki Ueda; **Kayoko Saito M.D.;** Junko Fujitani M.D., Reiko Arakawa M.D.; Kozue Takano; Ophthalmologist: Shigeko Yashiro M.D.; Physiotherapist: Maiko Seki; **Nozomi Sano M.D.;** Ophthalmologist: Akinori Uemura; Physiotherapists: Koji Fukuyama, Yuki Matsumoto, Hirofumi Miyazaki; **Minoru Shibata M.D.;** Physiotherapists: Kyoko Kobayashi, Yukie Nakamura; **Yasuhiro Takeshima M.D.;** Physiotherapist: Moe Kuma; *Poland (Part 2)*: **Anna** **Kostera-Pruszczyk M.D.,** Anna Fraczek M.D., Maria Jedrzejowska M.D., Anna Lusakowska M.D.; Ophthalmologists: Agnieszka Czeszyk-Piotrowicz, Wojciech Hautz, Klaudia Rakusiewicz; Physiotherapists: Malgorzata Burlewicz, Zuzanna Gierlak-Wojcicka, Malwina Kępa, Adam Sikorski, Marcin Sobieraj; **Maria** **Mazurkiewicz-Bełdzińska M.D.,** Anna Lemska M.D., Sandra Modrzejewska M.D.; Ophthalmologists: Mateusz Koberda, Urszula Stodolska-Koberda, Agnieszka Waśkowska; Physiotherapists: Jagoda Kolendo, Agnieszka Sobierajska-Rek; **Barbara Steinborn M.D.;** Ophthalmologists: Magdalena Dalz, Julia Grabowska, Wojciech Hajduk, Justyna Janasiewicz-Karachitos, Monika Klimas, Marcin Stopa; Physiotherapists: Ewa Gajewska, Beata Pusz; *Russia (Part 2)*: **Dmitry Vlodavets M.D.,** Evgenia Melnik M.D.; Ophthalmologists: Natalya Leppenen, Nataliya Yupatova; Physiotherapists: Anastasya Monakhova, Yulia Papina, Olga Shidlovsckaia; *Serbia (Part 2)*: **Vedrana** **Milic Rasic M.D.,** Vesna Brankovic M.D., Ana Kosac M.D., Ophthalmologists: Olivera Djokic, Vesna Jakšić, Ana Pepic; Physiotherapists: Jelena Martinovic; *Spain (Part 2)*: **Francina Munell Casadesus M.D.,** Eduardo Tizzano M.D.; Ophthalmologists: Charlotte Wolley Dod, Nieves Martín Begué, Olaia Subira; Physiotherapists: Bernat Planas Pascual, Esther Toro Tamargo; **Marcos Madruga Garrido M.D.;** Physiotherapists: José David Medina Romero, Marta Peña Salinas; **Andrés Nascimento Osorio M.D.;** Ophthalmologists: Ana Díaz Cortés, Enrique Jiménez Gañan, Hugo González Valdivia M.D., Simone Dowon Suh; Physiotherapists: Julita Medina Cantillo, Obdulia Moya, Nuria Padros, Sandra Roca Urraca; **Samuel Pascual Pascual M.D.;** Ophthalmologists: Sofía de Manuel, Susana Noval Martin; Physiotherapists: Paul Burnham, Sandra Espinosa Garcia, Mercedes Martinez Moreno; *Turkey (Part 2)*: **Haluk Topaloglu M.D., Ibrahim Oncel M.D.,** Nesibe Eroglu Ertugrul M.D., Bahadir Konuskan M.D., Neslihan Bilgin M.D., Seher Sari M.D.; Ophthalmologists: Bora Eldem M.D., Sibel Kadayıfçılar M.D.; Physiotherapists: Ipek Alemdaroglu, Aynur Ayse Karaduman, Oznur Tunca Yilmaz; *USA (Part 2)*: **Claudia Chiriboga M.D.,** Ophthalmologist: Steven Kane; Physiotherapists: John Lee, Donnielle Rome-Martin; **John W. Day M.D.,** Ophthalmologists: Shannon Beres; Physiotherapists: Tina Duong, Richard Gee, Sally Young.

**Sponsor (Roche) Personnel**

Sabine Fuerst-Recktenwald M.D. (Clinical), Anne Marquet PhD. (Pharmaceutical Research and Early Development), Nicoletta Milani Mülhardt PhD. (Pharmaceutical Research and Early Development), Dylan Trundell MSc. (Patient-centred Outcomes Research).
